# The Herbst Clinics in America

**Published:** 1886-12

**Authors:** 


### THE HERBST CLINICS IN AMERICA.

REPORTED EXPRESSLY FOR THE INDEPENDENT PRACTITIONER.

(Continued from page 645.)

### CLINIC GIVEN WEDNESDAY, JULY 28TH, AT THE NEW YORK COLLEGE OF DENTISTRY.

Dr. Herbst filled for Dr. Batterman the mesial surfaces of the right and left lower centrals. For a matrix he employed the thin piece of watch-spring previously described. He also explained and exhibited the general principles of the application of matrices.

*This is undoubtedly the case with dogs' teeth, which have a very large foramen. Further experiments may be necesssary to prove that it is also true of human teeth.—*Rep.*

CLINIC GIVEN THURSDAY, JULY 29TH, AT THE OFFICE OF DR. C. F. W. BÖDECKER.

Mr. M—— was the patient for whom Dr. Herbst contoured a right upper central incisor, the cavity involving the mesial surface, including the cutting edge. A matrix was made of shellac, into which two pieces of steel spring were inserted, one corresponding to the mesial surface, the other to the cutting edge of the cavity. The introduction of the gold occupied twenty-two minutes. Dr. J. M. Welch, of St. Paul, Minn., took the chair, for whom Dr. Herbst filled a left upper central in the distal surface, and a left upper lateral in the mesial surface. There were present at this clinic Drs. E. P. Brown, J. M. Welch, and C. F. W. Bödecker.

CLINIC GIVEN SATURDAY, JULY 31ST, AT THE OFFICE OF DR. C. F. W. BÖDECKER.

Dr. Herbst filled for Dr. Bödecker a right lower second molar. The cavity, which was prepared July 29th by Dr. E. P. Brown, involved the mesial and grinding surfaces. The dentine was found to be extremely sensitive, when the Herbst obtunder was applied with success. Upon the sensitive part of the dentine, near the cervical border of the cavity, a thin layer of tin was placed at the request of the patient, which has proved very successful in preventing the pain caused by thermal changes. The introduction of the gold occupied twenty-seven minutes. There were present at this clinic Drs. Wm. H. Atkinson and G. A. Mills.

CLINIC GIVEN MONDAY, AUGUST 2D, AT THE MEETING OF THE AMERICAN DENTAL ASSOCIATION AT NIAGARA FALLS.

Dr. Herbst filled for Dr. J. S. Marshall, of Chicago, a right lower second molar on the grinding surface, and a right lower second bicuspid on the distal and grinding surfaces, using for the latter operation the German-silver band matrix.

Dr. Herbst then attempted to fill for Dr. —— an upper central incisor, the cavity occupying the labial surface of the tooth. The rubber dam was applied, which was held above the cavity by Dr. Herbst's clamp, made for that purpose, but in this instance it proved unreliable, as the next tooth was an artificial crown, and the metal strip, which was pushed between the teeth, gave away during the introduction of the gold, when saliva entered the cavity and prevented the completion of the operation.

All the specimens and little inventions of Dr. Herbst, which have been previously described, were exhibited and highly appreciated by every one.

CLINIC GIVEN TUESDAY, AUGUST 3D, AT THE SAME PLACE.

Dr. Herbst filled for Mrs. H—— a right lower second bicuspid, the cavity occupying the distal and grinding surfaces. In this patient's mouth there were also two very large fillings inserted by Dr. Herbst at previous clinics, viz., a left lower second bicuspid filling, occupying the distal and grinding surfaces, and a right lower canine, involving one-third of the mesial, one-third of the distal, and one-eighth of an inch of the cutting surface of the tooth. These fillings were carefully examined by many and found perfect.

CLINIC GIVEN FRIDAY, AUGUST 6TH, AT THE OFFICE OF DR. C. F. W. BÖDECKER.

Dr. Bödecker built up for Mrs. W—— a right lower central incisor. The tooth had been ground away to the edge of the gum in front by its antagonist in the upper jaw, but the lingual wall, with the exception of about one-eighth of an inch of the cutting edge, was left intact. Before the cavity was excavated a matrix of shellac was prepared, to which three pieces of steel spring were adjusted, one on each side of the proximate surface and one over the cutting edge of the tooth to be filled. After the excavation of the cavity and the application of the rubber dam, the contour was restored with gold. In the first layers No. 0 Wolrab cylinders were used, while the rest was ~~built up~~ with No. 30 rolled gold, taking care to roughen and carefully examine every layer before another was introduced. The introduction of the gold occupied about an hour. The filling, when finished, was perfect and solid. The gentlemen present were Drs. Herbst, Wm. Carr and Degenhard.

After this filling was finished, Dr. Herbst filled for Dr. Ch. Degenhard a right lower molar, the cavity occupying the cervico-buccal portion of the tooth.

(TO BE CONCLUDED.)

